# Penguins perceive variations of source- and filter-related vocal parameters of species-specific vocalisations

**DOI:** 10.1007/s10071-023-01806-w

**Published:** 2023-07-04

**Authors:** Francesca Terranova, Luigi Baciadonna, Chiara Maccarone, Valentina Isaja, Marco Gamba, Livio Favaro

**Affiliations:** 1 Department of Life Sciences and Systems Biology, University of Turin, Turin, Italy; 2 Fondazione Zoom, Cumiana, Turin, Italy

**Keywords:** African penguin, Perception, Contact calls, Habituation-dishabituation paradigm, Source-filter theory

## Abstract

Animal vocalisations encode a wide range of biological information about the age, sex, body size, and social status of the emitter. Moreover, vocalisations play a significant role in signalling the identity of the emitter to conspecifics. Recent studies have shown that, in the African penguin (*Spheniscus demersus*), acoustic cues to individual identity are encoded in the fundamental frequency (*F*_0_) and resonance frequencies (formants) of the vocal tract. However, although penguins are known to produce vocalisations where *F*_0_ and formants vary among individuals, it remains to be tested whether the receivers can perceive and use such information in the individual recognition process. In this study, using the Habituation-Dishabituation (HD) paradigm, we tested the hypothesis that penguins perceive and respond to a shift of ± 20% (corresponding to the natural inter-individual variation observed in ex-situ colonies) of *F*_0_ and formant dispersion (ΔF) of species-specific calls. We found that penguins were more likely to look rapidly and for longer at the source of the sound when *F*_0_ and formants of the calls were manipulated, indicating that they could perceive variations of these parameters in the vocal signals. Our findings provide the first experimental evidence that, in the African penguin, listeners can perceive changes in *F*_0_ and formants, which can be used by the receiver as potential cues for the individual discrimination of the emitter.

**Supplementary Information:**

The online version contains supplementary material available at 10.1007/s10071-023-01806-w.

## Introduction

A crucial aspect in the study of animal vocal communication is the identification of the acoustic parameters that may encode salient information for the receiver (Owings and Morton 1998; Seyfarth and Cheney 2010). The application of the source-filter theory for human voice production (Fant 1980) to nonhuman animals’ vocalisations (Beckers et al. 2004; Taylor and Reby 2010; Budka and Osiejuk 2013; Vannoni and McElligott 2008) and the use of vocal tract modelling approaches (Gamba et al. 2017; Reby et al. 2018) have advanced our knowledge of the use of source-and filter-related vocal parameters to convey indexical and individual identity information in bird and mammal vocal signals. In penguins, the source-filter theory approach allowed a better understanding of the vocal repertoire of the African penguin (*Spheniscus demersus*) and the role of the independent contribution of the different organs of the respiratory system on vocal production (lung—duration and temporal patterns; syrinx—source, determining the fundamental frequency; the vocal tract—filter, generating formant frequencies) (Favaro et al. 2015). Moreover, recent studies have demonstrated that in the African penguin, at least two vocal types, namely the contact calls and the Ecstatic Display Songs (EDS), encode information on the individual identity of the emitter (Favaro et al. 2016, 2017). In these vocal types, the fundamental frequency (*F*_0_) and the formants are essential cues to assign the identity of the callers. In addition, based on the relative stability of contact calls and EDS, it is possible to identify individuals reliably over consecutive breeding seasons (Calcari et al. 2021).

Considering the relative importance that vocalisations play in penguins’ social life, such as group cohesion, mitigation of conflicts and recognition between mates, parents, and offspring, it is reasonable to expect that penguins can attend to temporal (duration, rhythm), source-related (*F*_0_), and filter-related (formants) parameters encoded in their calls (Lengagne et al. 1997; Aubin and Jouventin 2002a; Jouventin and Aubin 2002; Favaro et al. 2015; Jouventin and Dobson 2018). Indeed, previous studies have shown that non-nesting penguins of the genus *Aptenodytes* attend to the beats generated by the interaction of the two fundamental frequencies produced in their syrinx (i.e., two-voice system) to infer the individual identity of conspecifics (Aubin et al. 2000), while, nesting species, such as the Adélie (*Pygoscelis adeliae*) or the Gentoo (*P. papua*) penguins, pay more attention to the spectral profile and the precise frequency values of the harmonics (Aubin and Jouventin 2002b). These differences in attending to *F*_0_ and formants of the vocalisations have evolved in response to different breeding systems (non-nesting vs nesting), socio-ecological pressure, and the level of recognition needs across the different species (Jouventin and Aubin 2002). Even though it has been shown that source-and filter-related parameters encode meaningful biological information in penguin species, the investigation of the perceptual and functional relevance of these vocal parameters from the listeners’ perspective is lacking and unexplored in the African penguin.

The investigation of the receiver's ability to recognize an individual as unique can reveal important aspects of communicative abilities on colonial life. Here, by using re-synthesised contact calls of different African penguins in combination with the habituation-dishabituation paradigm, we tested the hypothesis that African penguins perceive and respond to a shift in the *F*_0_ and formant dispersion (ΔF) of their vocalisations within the species-specific variability of these parameters. The HD paradigm is a powerful behavioural paradigm investigating perceptual abilities in nonhuman animals (Rendall et al. 1996; Charlton et al. 2011a; Baciadonna et al. 2019; Carlson et al. 2020). This paradigm estimates the ability to discriminate whether two stimuli are perceived differently based on the behavioural responses they elicit. When a stimulus is presented continuously, the subject’s attention towards it declines (habituate). Instead, when a new stimulus is presented, the subject's attention is renewed if the stimulus is perceived as different from the previous one (dishabituate). In detail, we predicted that after a reduced response to conspecific contact calls, penguins would show a renewed response when the fundamental frequency or formant dispersion is increased or decreased by 20% from their original frequency values. If confirmed, our study will enhance our understanding on the penguins’ perception of the information encoded in the spectral envelope of their species-specific vocalisations, demonstrating that source- and filter-related acoustic parameters likely play a crucial role for individual recognition.

## Methods

### Subjects

This study was conducted at Zoom Torino (Italy) between February 2021 and April 2022. At the beginning of the study, the colony consisted of 37 adult penguins born in four different zoological facilities in Europe (Artis Royal Zoo, Amsterdam, NL; Bird Park Avifauna, Alphen an den Rijn, NL; Wilhelma Zoo, Stuttgart, DE; South Lake Wild Animal Park, Manchester, UK). However, during the study period, the number of penguins in the colony changed. In March 2021, 23 penguins were relocated, while between August and October 2021 a group of 16 penguins was added to the colony (from Zoo Wrocław, Wrocław, PL; Safari de Peaugres, Lyon, FR) bringing the total number to 30 penguins. Overall, a total of 14 adult African penguins (eight males and seven females, age range: 1–34 y.o.) were tested. Two penguins passed away during data collection and could not be tested in all experimental conditions.

### Acoustic stimuli

We selected the acoustic stimuli from a database of 120 contact calls belonging to 20 individuals (10 males and ten females) from two non-familiar penguin colonies housed at Zoomarine (Pomezia, Italy) and Zoological Garden of Pistoia (Pistoia, Italy). We collected the acoustic data from the Zoomarine colony between February and October 2020 (7.00–13.00; 44 days; 220 h total). We recorded at the Zoological Garden of Pistoia between October 2016 and June 2017 (8.00–18.00; 68 days; 230 h total). We collected all recordings between 3 to 10 m from the vocalizing individuals with a RODE NTG-2 shotgun microphone (flat frequency response 20 Hz to 20 kHz, max SPL 131 dB) connected to a ZOOM H5 handy recorder (48 kHz sampling rate). We saved audio files in WAV format (16-bit amplitude resolution) and stored them on a secure digital memory card.

### Playback sequences and procedures

We used the HD paradigm to investigate whether penguins could perceive and discriminate conspecific contact calls when the *F*_0_ and ΔF were modified one at a time (Carlson et al. 2020). This paradigm habituates an individual to a stimulus through repeated stimulus presentation (reduction of responses). The presentation of a dishabituation stimulus follows habituation. If the individual pays attention to the new stimulus, this would indicate an ability to perceive and discriminate the change between the two stimuli. Furthermore, this paradigm provides a control condition (i.e., rehabituation) to exclude the possibility that the dishabituation reaction was not simply due to a spontaneous recovery of the pre-habituation level. This control is achieved by presenting a stimulus previously encountered during the habituation phase after the dishabituation phase (Charlton et al. 2011a; Baciadonna et al. 2019).

**Fig. 1:**
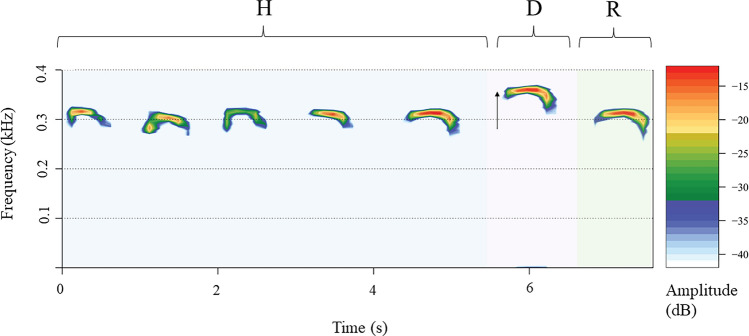
Sequence of contact calls used in a playback experiment, shift *F*_0_ + 20%. *H* Habituation phase, *D* Dishabituation phase, *R* Rehabituation phase. The arrow indicates the call with *F*_0_ increased by 20%. Figure created with the R package ‘seewave’ v. 2.2.0 (Sueur et al. 2008). Spectrogram parameters: window length = 10,000, overlap = 60, window type = ‘Hann’

For each non-familiar donor individual (N = 20), we selected six good-quality contact calls with low background noise (a total of 120 contact calls) to build 40 playback sequences. Each sequence comprised seven contact calls separated by a time interval of 15 s. Four calls from the donor sequence were selected, concatenated randomly, and used to build the habituation phase (H1–H4) and build two playback sequences. We used each donor's other two contact calls as the unique and last contact call of the habituation phase (H5), which we concatenated to the contact calls H1–H4 of the sequences. The dishabituation phase (D, call 6) was created by modifying the last call of habituation (H5). Although call D was constructed using H5, the manipulation of the acoustic parameters led to an entirely unrelated novel acoustic stimulus (Fig. 1). It is still possible that the penguin does not perceive the shift in acoustic parameters, and thus, in line with the assumptions of the HD paradigm, repeated exposure to the same stimulus will lead to the habituation. To shift the fundamental frequencies, we calculated, using Praat v. 6.1.5 (Boersma and Weenink 2022), the fundamental frequency (*F*_0_ mean) of the H5. Subsequently, the *F*_0_ mean of H5 was increased or decreased by 20% by using the *Convert* >  > Change *gender function* >  > *New pitch median* (Hz) tab in Praat and saved as a new mono file in wav format (16-bit resolution). We chose the 20% variation in the fundamental frequency and formants considering the inter-individual variation of *F*_0_ and ΔF observed in the contact calls of *ex-situ* African penguin colonies (Favaro et al. 2015).

We used the formant shift ratio tab on Praat to increase or decrease the ΔF by inserting 1.2 or 0.8 values. Finally, for the rehabituation phase (R, calls 7), we concatenated the last call used in the habituation phase (H5) to the playback sequences. The original duration of the calls was left unchanged, and we equalised the peak amplitude of calls during the preparation of playback sequences. We broadcast playback sequences from a Bose® Soundlink Mini II loudspeaker connected wirelessly to an Oppo® A72 smartphone at an approximately natural amplitude (72.40 ± 2.47 dB) measured at 1 m using a Monacor® SM-2 sound level meter.

We presented four different playback sequences for each tested penguin: two for each acoustic parameter (*F*_0_ and ΔF) shifted + 20% and − 20%. Before the experiment started, the experimenter inspected each nest available in the colony. When we found a penguin in the nest, the experimenter identified the subjects by using a coloured flipper band located on the wings. After the identification, the experimenter placed a video camera Sony® (HDR-CX140) and the speaker (aligned) 5 m away from the nest and moved away from the penguin’s view. After approx. 3–5 min, the experimenter played the acoustic sequence selected for the subjects remotely. At the end of the playback sequences, the experimenter approached the nest to remove the camera and the speaker. Each subject was never tested more than twice on the same day, and we allocated a minimum of 1 h break between each playback presentation.

### Scoring of the behavioural responses

The duration of the first looking (s) at the speaker and latency (s) were measured. We defined latency as the amount of time that elapses between the onset of the stimulus and any head movement towards the speaker. We assigned a maximum time of 15 s for the latency for subjects that did not respond. We defined the duration of first looking as the time the penguin looked at the speaker from the end of the latency until any head movement occurred. Without latency, this behaviour was not scored, including when the penguin was already directed toward the speaker. Subjects’ responses for each call included in each playback sequence were analysed using BORIS v. 7.10.7 (Friard and Gamba 2016). We tested the reliability of the parameters measured, scoring 20% of the sessions to test them by the two observers. The interclass correlation coefficient calculated for all the behaviours analysed statistically was: 0.84 for the duration of first looking and 0.84 for latency.

### Statistical analyses

We used the software R version 4.1.0 (R Development Core Team 2021) for statistical analyses. The first step was to establish whether penguins habituated to the sounds by comparing the duration of the first look and latency of the five calls (H1–H5) played during the habituation phase.

Subsequently, in the discrimination phase, the last habituation call (H5) was compared with the dishabituation call (D), and the duration of the first look to the dishabituation call was compared to the rehabituation call (R) to establish whether penguins were able to detect any differences between these calls.

The duration of the first look was analysed using a Generalised Linear Mixed Model (GLMM) using the lme4 package (Bates et al. 2015). The model included the duration of the first look (log-transformed) as the response variable, Phase (H1 to H5 for the habituation phase and H5, D and R for the discrimination phase), Condition (Fundamental frequency and Formants), and Shift frequencies (− 20% and + 20%) as fixed factors. We assessed the significance of the full model by comparing this model with the model that included only the random factors (null model) using a likelihood ratio test. We checked the model fit and over‐dispersion using the DHARMa 0.3.3.0 package (Hartig 2020). The p-value of each factor was derived using the “drop1” function (Barr et al. 2013). Also, the subjects’ identity was included as a random factor to control for repeated measurements of the same subject in all models performed. Finally, we performed pairwise comparisons using the lsmeans multiple contrast package (Lenth 2016) with a Tukey post hoc test.

We analysed latency with Cox proportional hazards models with the function *coxme* in the R package Survival. The model included latency as the response variable, Phase (H1 to H5 for the habituation phase and H5, D and R for the discrimination phase), Condition (Fundamental frequency and Formants), and Shift frequencies (− 20% and + 20%) as fixed factors. Also, the subjects’ identity was included as a random factor to control for repeated measurements of the same subject in all models performed. The p-value of each factor was derived using the ANOVA function followed by a Tukey post hoc test to account for pairwise comparisons. We deemed subjects that did not respond with 15 s as censored results.

## Results

**Fig. 2:**
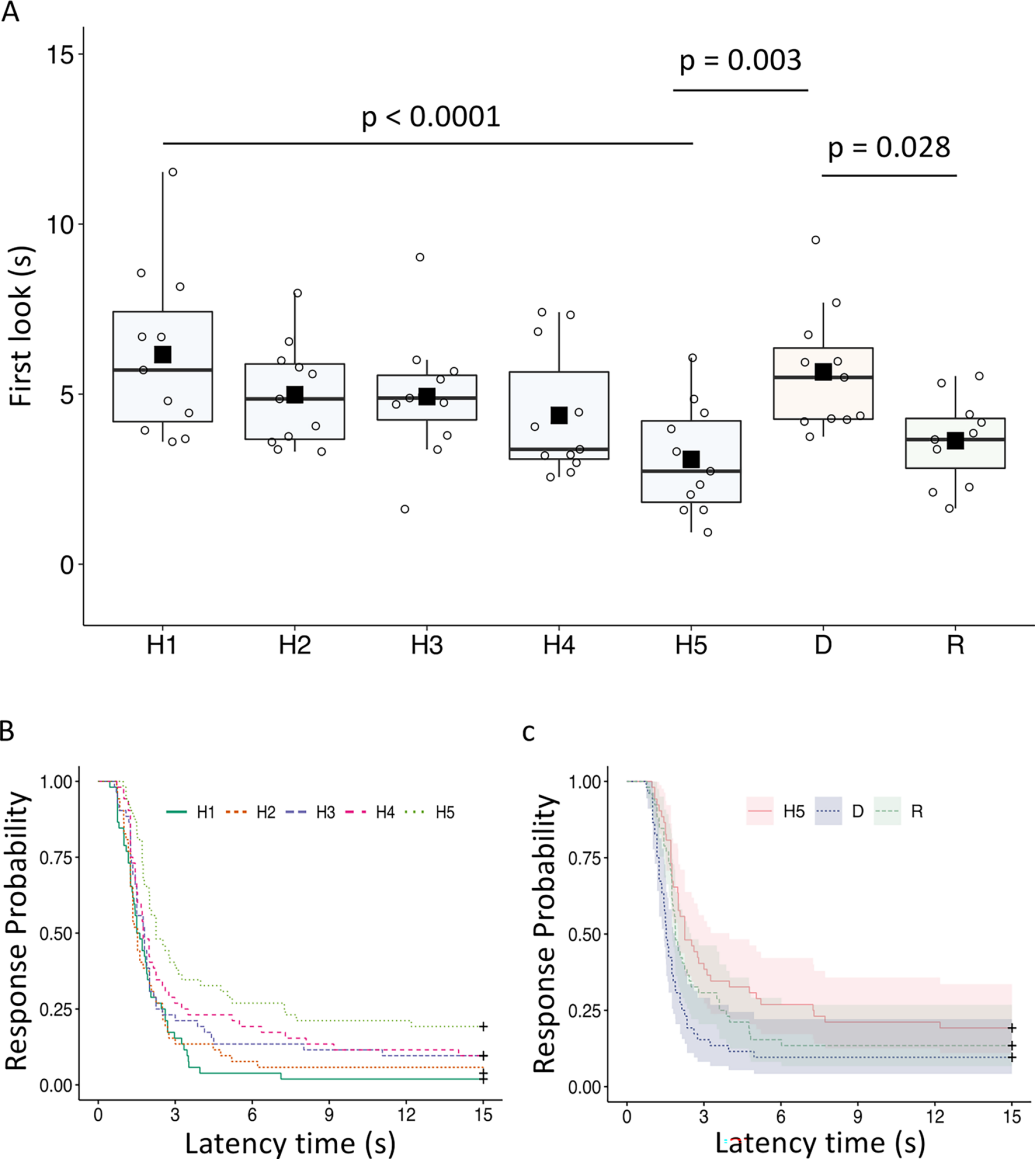
Behavioural response of penguins during playback experiments. **A** Duration of the first look to the habituation–dishabituation playback sequences (N = 14). The box plots presented here (which illustrate horizontal lines = median; black squares = mean; boxes extend from lower to upper quartile, and whiskers indicate interquartile range above the upper quartile (max) or below the lower quartile (min), show an initial diminution of response levels across the habituation phase (H1–H5) followed by a renewal of response levels to the dishabituation stimulus (D). Finally, a decrease in response levels after the rehabituation stimulus, returning to that of the last playback of the habituation phase (H5); **B** probability during the habituation phase of penguins to respond at the playback calls; **C** probability during the H5, D and R calls of penguins to respond at the playback

During the habituation phase, the duration of looking at the playback calls gradually decreased (Table 1). Post-hoc analyses revealed that the duration of the first look at call H1 (mean ± SE = 6.27 ± 0.61 s) was significantly longer compared to the duration of the first look at call H5 (mean ± SE = 3.33 ± 0.44 s; estimate = − 0.60, s.e. = 0.12, z = − 4.74, p < 0.001). Neither condition nor shifted *F*_0_ and ΔF predicted the duration of the first look during habituation (Table 1). The duration of the first look was predicted by phase (Table 1) when H5, D and R were considered (Table 1, Fig. 2A). Penguins significantly increased the duration of looking between the dishabituation playback (D; mean ± SE = 5.43 ± 0.48 s) and the last playback of the habituation phase (H5; estimate = 0.45, s.e. = 0.13, z = 3.26, p = 0.003; Fig. 2A). In addition, penguins significantly reduced the duration of the first look between the rehabituation playback (R; mean ± SE = 3.65 ± 0.44 s), and the dishabituation call (D; estimate = − 0.35, s.e. = 0.13, z = − 2.55, p = 0.028; Fig. 2A), with a similar first-look duration of the last habituation playback (H5). Neither condition nor shifted *F*_0_ and ΔF predicted the duration of the first look during habituation (Table 1).

**Table 1:** Summary of the GLMM for the duration of looking

Response variable	Fixed factor	Estimate	s.e	*t*	*p*-value
Duration first look					
	(intercept)	1.81	0.12		
	Phase				
	H2	− 0.17	0.12	− 1.39	< 0.0001
	H3	− 0.23	0.12	− 1.85	
	H4	− 0.37	0.12	− 2.19	
	H5	− 0.60	0.12	− 4.74	
	Condition	− 0.02	0.08	− 0.34	0.73
	Shift	0.007	0.08	0.09	0.92
Duration first look					
	(intercept)	1.22	0.13		
	Phase				
	D	0.45	0.13	3.26	0.003
	R	0.09	0.13	0.71	
	Condition	− 0.11	0.11	− 0.97	0.32
	Shift	0.06	0.11	0.56	0.57

Upper half: GLMM examining the influence of the fixed factors on the response variables during the habituation phase. Results of the reduced model when the duration of looking was considered (full vs. null: *X*^2^_(6)_ = 24.07, p < 0.0001). Phase predicted duration of first look, and neither condition nor shift predicted the duration of first look (in seconds). Lower half: GLMM examining the influence of the fixed factors on response variables when comparing H5, D and R. Results of the reduced model when the duration in proximity was considered (full versus null: *X*^2^_(4)_ = 12.48, p = 0.014). Phase predicted the duration of the first look and neither condition nor shift predicted duration (in seconds) of the first look

During the habituation phase, latency to look at the playback calls gradually increased (Table 2, Fig. 2B). Post-hoc analyses revealed that the latency at call H1 (mean ± SE = 2.12 ± 0.29 s) was significantly shorter compared to the latency at call H5 (mean ± SE = 5.17 ± 0.72 s; estimate = − 0.87, s.e. = 0.21, z = − 4.12, p < 0.001). Neither condition nor shifted *F*_0_ and ΔF predicted latency during habituation (Table 2, Fig. 2A). The latency to look at playback calls was predicted by phase (Table 2) when H5, D and R were considered (Table 2, Fig. 2C).

**Table 2:** Summary of the Cox proportional hazards models for the latency

Response variable	Fixed factor	Coef	Exp (coef)	Se (coef)	*z*	*p*-value
Latency						
	Phase					
	H2	− 0.08	0.91	0.20	− 0.44	0.0002
	H3	− 0.31	0.72	0.20	− 1.55	
	H4	− 0.50	0.60	0.20	− 2.44	
	H5	− 0.87	0.41	0.21	− 4.12	
	Condition	− 0.04	0.95	0.13	− 0.30	0.76
	Shift	0.13	1.14	0.13	1.02	0.30
Latency						
	Phase					
	D	0.75	2.12	0.21	3.47	0.002
	R	0.28	1.32	0.21	1.31	
	Condition	− 0.06	0.94	0.17	− 0.34	0.73
	Shift	− 0.07	0.92	0.17	− 0.42	0.67

Upper half: Cox proportional hazards models examining the influence of the fixed factors on the response variables during the habituation phase. Phase predicted the response latency (in seconds) probability to respond at the playback calls (*X*^2^_(4)_ = 21.21, p = 0.0002), and neither condition (*X*^2^_(1)_ = 0.09, p = 0.76), nor shift (*X*^2^_(1)_ = 1.04, p = 0.30) influenced the response latency probability. Lower half: Cox proportional hazards models examining the influence of the fixed factors on response variables when comparing H5, D and R. Phase predicted the response latency (in seconds) probability to respond at the playback calls (*X*^2^_(2)_ = 12.31, p = 0.002), and neither condition (*X*^2^_(1)_ = 0.11, p = 0.73), nor shift (*X*^2^_(1)_ = 0.17, p = 0.67) influenced the response latency probability

Penguins looked faster between the dishabituation playback (D; mean ± SE = 2.96 ± 0.55 s) and the last playback of the habituation phase (H5; estimate = 0.75, s.e. = 0.21, z = 3.46, p = 0.0015; Fig. 2C). The latency to look at the playback calls between the rehabituation playback (R; mean ± SE = 3.95 ± 0.62 s) and the dishabituation call was longer, despite the result being not significant (D; estimate = − 0.47, s.e. = 0.21, z = − 2.18, p = 0.073, Fig. 2C). Neither condition nor shifted *F*_0_ and ΔF predicted the duration of the first look during habituation (Table 2).

## Discussion

The study provides convincing evidence that African penguins attend to *F*_0_ and ΔF of their species-specific vocalisations. Penguins showed a significant renewal of response (i.e., duration of first looking) when hearing the contact calls in which *F*_0_ or the ΔF had been shifted from their initial frequencies during the habituation phase. The significantly reduced response to the rehabituation phase indicates that the response to the shifted contact calls was not simply a recovery of the pre-habituation response level. Therefore, our findings demonstrate that penguins perceive and respond to changes in fundamental frequency and formant dispersion within the natural range of variation and that these differences in shift (± 20%) were enough to be detected. Furthermore, the spontaneous penguins’ responses (i.e., in the absence of training) suggest the functional significance of *F*_0_ and formants for the vocal communication system in the African penguin.

Although contact calls are one of the most common type of calls used in a variety of animal species and most likely have evolved primarily to maintain group cohesion (Kondo and Watanabe 2009), a growing body of evidence has suggested that contact calls can be used in individual recognition, especially in fission–fusion social systems in which small group of individuals disperse during the foraging activity and later aggregate in larger groups (Macedonia 1986; Mathevon 1997; Wanker and Fischer 2001; Sharp and Hatchwell 2005; Buhrman-Deever et al. 2008, Mumm et al. 2014). Similarly, in the African penguin, contact calls are emitted to maintain group cohesion when visually isolated from other individuals (Favaro et al. 2015, 2016), often when foraging at sea (McInnes et al. 2020). Furthermore, in *ex-situ* colonies, it is common to observe adult penguins emitting contact calls to keep contact specifically with their partner (Baciadonna et al. 2021, 2022) or juveniles uttering these vocalisations in the presence of the keepers (F.T. personal observation). Beyond the African penguins, recent findings have provided convincing evidence that the fundamental frequency of the contact calls play a role also for colony-mate recognition in different animal species: birds (*Forpus passerines*; Berg et al. 2011), cervids (adult female of fallow deer; Torriani et al. 2006), bats (*Antrozous pallidus*; Arnold and Wilkinson 2011).

The ability of penguins to detect slight variations in the *F*_0_ and formants of manipulated contact calls further emphasizes the significance of these acoustic parameters in conveying biologically meaningful information across a wide range of animal taxa, even those that are phylogenetically distant (Taylor & Reby 2010). Previous studies demonstrated that humans are sensitive to frequency spacing shifts of about 4% in speech (Smith et al. 2005; Puts et al. 2007; Monahan and Idsardi 2010), while non-human mammals like the red deer (Charlton et al. 2007a) and the giant panda (Charlton et al. 2010) can perceive shifts of 5–10% in their species-specific calls. Our results showed that the African penguins can detect a shift in *F*_0_ and formant spacing of 20%. However, a possible limitation of this study is the lack of determining a precise threshold sensitivity of African penguins to the variation of these vocal parameters. Further studies are needed to investigate these aspects in detail.

The duration of the first look toward the speaker suggests that penguins were equally attentive regardless of conditions (*F*_0_ or ΔF) and the type of manipulation applied (± 20%) to the dishabituation stimulus. Penguins’ latency across the five calls increased as expected regardless of the conditions and the type of re-synthesised signal considered indicative that they gradually habituated. In addition, penguins responded significantly faster to the dishabituation call regardless of the conditions and the shift we applied. By contrast, penguins’ response to the rehabituation call was not different from the last call of habituation, although the mean latency decreased. According to the habituation-dishabituation paradigm, this response indicates that these two calls were different enough to be perceived by the penguins. Conforming to the methodological paradigm to validate the robustness of behavioural responses that occurred during the presentation of the dishabituation calls, we should expect a similar behavioural response (i.e., mean latency) observed in the last call of habituation. According to our prediction, the unexpected behavioural pattern observed would suggest that the response to the dishabituation calls was simply a spontaneous rebound of pre-habituation response levels. Although this is plausible, we cannot exclude other possible explanations, especially because penguins' latency to react between the rehabituation playback and the dishabituation call was longer, despite the results not being significant. It is also possible that latency is more indicative of general penguins' attention/alertness towards external stimuli, and if relevant, they might allocate more time, as we had observed when the duration of the first look was included. Further research is necessary to interpret this result in a broader perspective. For instance, measuring physiological parameters, such as the heart rate and the heart rate variability, could provide additional information about the responses of the penguins’ sympathetic and parasympathetic system during the playback of the dishabituation stimulus (Baciadonna et al. 2019).

Spontaneous perception of *F*_0_ or ΔF in species-specific vocalisations has been demonstrated in several mammals (Charlton 2007a; Charlton et al. 2008a, b; Charlton et al. 2010, 2011a; Ghazanfar et al. 2007; Reby et al. 2005; Fitch and Fritz 2006; Searby and Jouventin 2003) and a few bird species (Fitch and Kelley 2000; Vignal et al. 2004; for a review: Dooling et al. 2000), and quite often the targeting aspect investigated is the perception of formant shift. The emphasis on filter-related acoustic parameters in mammals is due to different reasons: first, they encode cues to individual identity (Rendall et al. 1998; Fitch 1997; McComb et al. 2003; Vannoni and McElligott 2007; Charlton 2011b; Green et al. 2019); second because they are honest cues to body size compared to the source-related parameters with implications in mate selection (Fitch 1997, 2000; Charlton et al. 2007b; Reby and McComb 2003; Vannoni and McElligott 2008; Taylor et al. 2010) and, finally because the formants play a role in vowel perception in human speech (Hillenbrand and Clark 2009; Root-Gutteridge et al. 2019). Investigating the perceptual and functional role of vocalisations in other species can help us to reconstruct the evolution of complex vocal communication system (Garcia and Favaro 2017; Fitch 2010).

Here, we provide evidence that penguins discriminate source- and filter-related components of contact calls which have been indicated in previous studies as the primary cues to individual identity in penguins of the genus *Spheniscus* (Favaro et al. 2015; 2016; 2017; Calcari et al. 2021). Further studies using a similar methodological paradigm are needed to investigate whether penguins can discriminate changes in other acoustic parameters potentially relevant to their body size and mass. These might include the duration of the vocal units and the composition and temporal patterns of the vocal sequences (Favaro et al. 2020). Playback experiments, especially in wild colonies, could also be relevant to determine how subjects use the source- and filter-related acoustic information in mate choice and intersexual competitions. In summary, our findings on penguins’ responses to variation in fundamental frequency and formants dispersion of species-specific calls suggest that these aspects play an essential evolutionary functional role in the animal communication system and pave the way for further comparative studies.

## Supplementary Information

Below is the link to the electronic supplementary material.

Supplementary file1 (MP4 46550 KB)

## Data Availability

Datasets generated and/or analysed during the current study are available from the corresponding author upon request.
